# The adjunctive application of transcranial direct current stimulation in the management of de novo refractory epilepsia partialis continua in adolescent‐onset *POLG*‐related mitochondrial disease

**DOI:** 10.1002/epi4.12094

**Published:** 2018-01-11

**Authors:** Yi Shiau Ng, Henriette van Ruiten, H. Ming Lai, Rebecca Scott, Venkateswaran Ramesh, Karen Horridge, Robert W. Taylor, Doug M. Turnbull, Gráinne S. Gorman, Robert McFarland, Mark R. Baker

**Affiliations:** ^1^ Wellcome Centre for Mitochondrial Research Institute of Neuroscience Newcastle University Newcastle United Kingdom; ^2^ Department of Paediatric Neurology Royal Victoria Infirmary Newcastle upon Tyne United Kingdom; ^3^ Department of Clinical Neurophysiology Royal Victoria Infirmary Newcastle United Kingdom; ^4^ Department of Paediatrics City Hospitals Sunderland NHS Foundation Trust Sunderland United Kingdom; ^5^ Institute of Neuroscience Newcastle University Newcastle upon Tyne United Kingdom

**Keywords:** Focal seizures, Mitochondrial disease, Neurostimulation, Refractory status epilepticus

## Abstract

Focal status epilepticus in *POLG*‐related mitochondrial disease is highly refractory to pharmacological agents, including general anesthesia. We report the challenges in managing a previously healthy teenager who presented with de novo epilepsia partialis continua and metabolic stroke resulting from the homozygous p.Ala467Thr *POLG* mutation, the most common pathogenic variant identified in the Caucasian population. We applied transcranial direct current stimulation (tDCS; 2 mA; 20 min) daily as an adjunctive therapy because her focal seizures failed to respond to five antiepileptic drugs at maximal doses. The electrical and clinical seizures stopped after 3 days of tDCS. The second course of tDCS was administered for 14 days when the focal seizures re‐emerged a month later. The patient tolerated the procedure well. Following 4 months of hospitalization and prolonged community rehabilitation, our patient has now returned to full‐time education with support, and there is no report of cognitive deficit. We have demonstrated the safety and efficacy of tDCS in treating refractory focal motor seizures caused by mitochondrial disease.

The clinical manifestations of mitochondrial disease are heterogeneous,^1^ and seizures affect approximately a quarter of adult patients.^2^ Refractory seizures have been long recognized as one presenting feature of Alpers‐Huttenlocher syndrome (AHS) in early childhood caused by pathogenic variants in the *POLG* gene. More recently, juvenile‐onset de novo status epilepticus with or without hepatic failure has been increasingly reported as a *POLG*‐related mitochondrial disorder.^3^, ^4^ Mixed seizure types, including generalized convulsive seizures, epilepsia partialis continua (EPC), myoclonus, and occipital seizures, are frequently observed.^5^ To date, there remains no effective treatment for *POLG*‐related epileptic encephalopathy. New therapeutic approaches are desperately needed, given the carrier frequency of common *POLG* mutations is prevalent (0.5–1% in the white European population) and high disease burden and mortality observed in those in whom disease presents with intractable epilepsy (median interval between disease onset and death was 1 year).^5^

We have directly managed two cases of juvenile‐onset *POLG* disease from our region over the last 8 years. The first case was a 17‐year‐old girl who died in the intensive care unit despite maximal medical intervention in refractory status epilepticus (for details see Data S1 and Figure S1). Here we describe details of the second case, in whom, despite a near fatal course, seizures stopped after applying a noninvasive brain stimulation technique known as transcranial direct current stimulation (tDCS).

## Case report

A right‐handed 15‐year‐old girl who was previously fit and well, presented with a prolonged generalized tonic‐clonic seizure that required intubation and sedation in June 2015. Extubation was performed 48 h later, and jerking of her left arm spreading periodically to involve the rest of her body was noted. Metabolic, structural, infective, and autoimmune causes of seizures were excluded with normal routine laboratory studies, MRI head, lumbar puncture, negative anti‐NMDAR and anti‐VGKC antibodies, and MRI pelvis. She was commenced on oral phenytoin and levetiracetam and discharged home after several days of admission. A month later, she represented with five generalized tonic‐clonic seizures, preceded by a 2‐day history of headache, positive visual phenomena (colored circles), unsteadiness, and hypersomnolence. Seizures were inadequately controlled with a combination of phenytoin, levetiracetam, sodium valproate, and pulses of methylprednisolone, and treatment was escalated to general anesthesia. Following extubation, the generalized seizures ceased, but she remained encephalopathic and had frequent focal motor seizures affecting the left arm. Electroencephalography (EEG) confirmed electrical EPC arising from the right occipital region. Sodium valproate was stopped as mitochondrial disease was suspected at this stage. Her liver function was normal.

**Figure 1:**
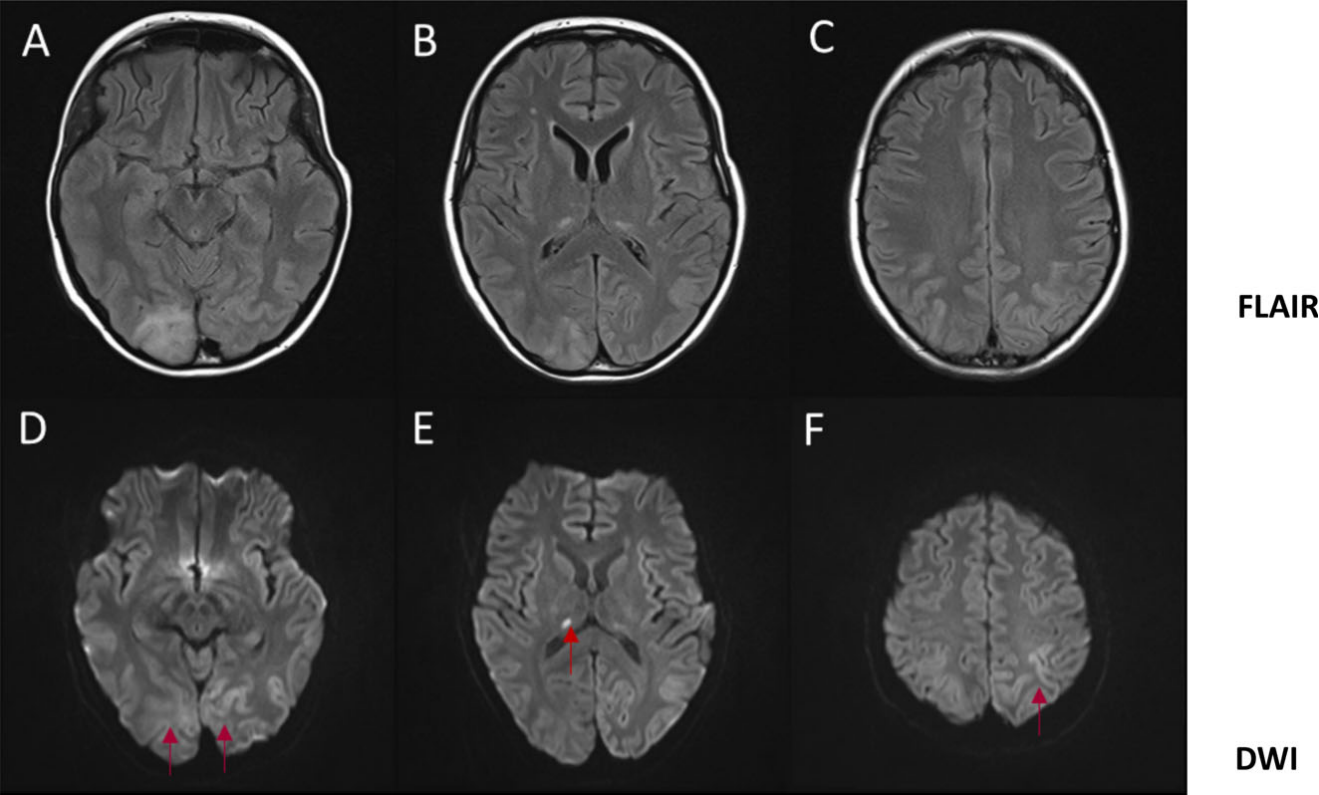
Axial view of MRI head. FLAIR‐sequence (A–C) shows hyperintense lesions involving the right occipital lobe, right thalamus, and bilateral parietal lobes. DWI sequence (D–F) shows restricted diffusion in the bilateral occipital lobes, right thalamus, and left parietal lobe (red arrows) with increased ADC map (not shown), suggestive of vasogenic edema.

The patient was transferred to the regional neurology unit. She developed a new epileptic focus affecting the right arm after transfer, and the MRI brain showed stroke‐like lesions (Figs. 1A–F). Direct sequencing of the *POLG* gene (GenBank Accession number NM_002693.2) identified a pathogenic homozygous c.1399G>A, p.(Ala467Thr) variant. Despite a cocktail of antiepileptic drugs (AEDs), including lacosamide, perampanel, phenobarbitone, phenytoin, levetiracetam, and diazepam, EPC did not abate. The patient was admitted to the pediatric intensive care unit where the head was cooled and thiopentone administered until a burst‐suppression pattern was achieved on EEG. Following the withdrawal of thiopentone, recovery of consciousness took 11 days. She was free of overt clinical seizures for several days but had ongoing electrographic focal status epilepticus and significant encephalopathy. Debilitating EPC reemerged, affecting the right arm, leg, and paraspinal muscles, and frequent myoclonic jerks (including abdominal muscles) developed two weeks later. Further EEG studies continued to detect epileptic discharges arising from the posterior quadrant of the right hemisphere that spread over to the left side at times; there was no apparent EEG correlate with right upper and lower limb clonic movements. These reemergent seizures were refractory to AED therapy. Repeat administration of thiopentone was not considered owing to the side effects of prolonged recovery and risk of cardiorespiratory depression.

Concern over the patient's deteriorating health urged us to consider cathodal tDCS as an adjunctive treatment.

## Methods for transcranial direct current stimulation

The clinical application of tDCS was approved as an emergency compassionate therapeutic intervention by the Newcastle upon Tyne Hospitals NHS Foundation Trust (NuTH) New Interventional Procedure Committee (NIPC) on a named patient basis. We obtained written consent from the patient's parents.

**Figure 2:**
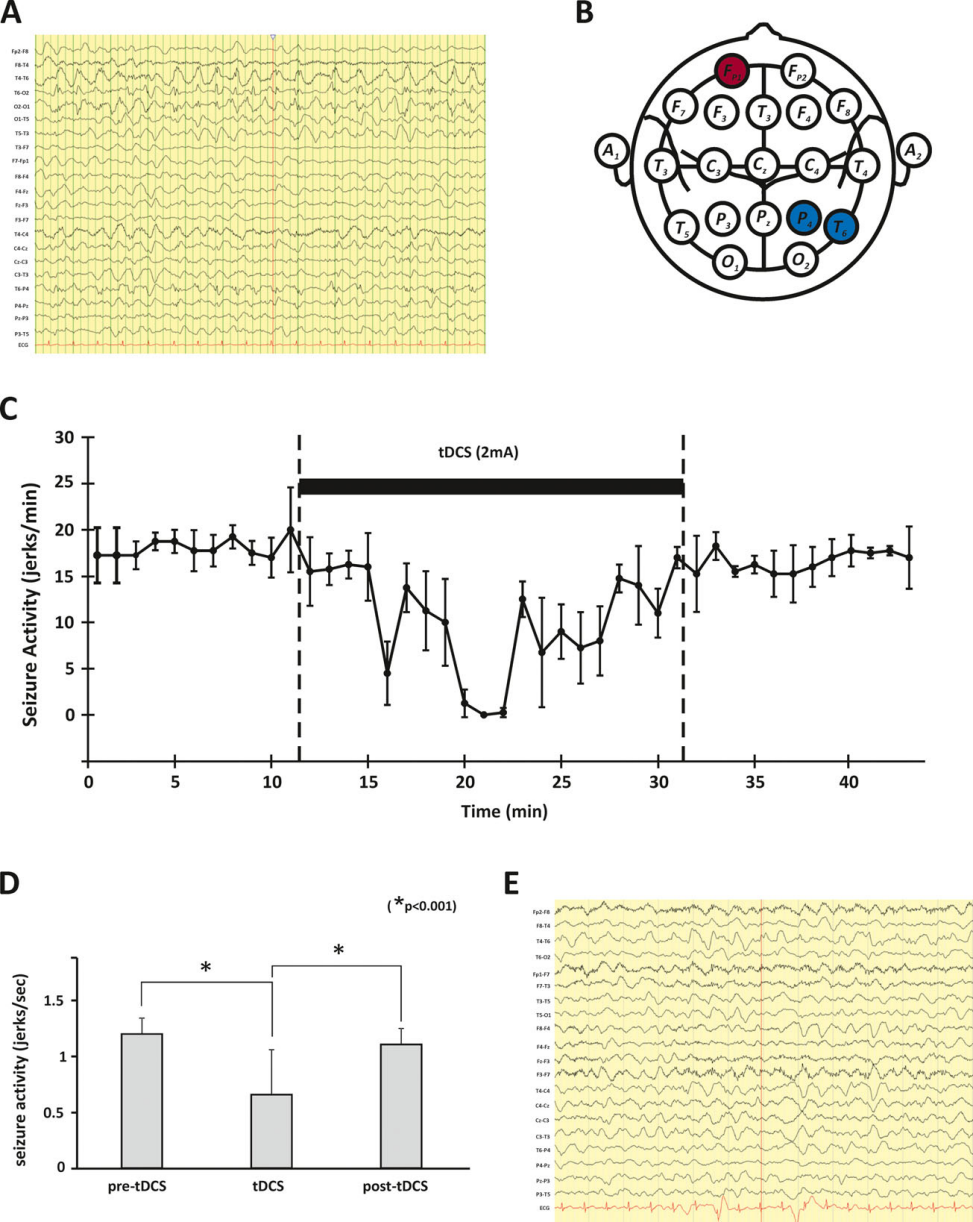
Electrophysiology. Pretreatment EEG (A) showing continuous seizure activity over the right parieto‐tempero‐occipital region. Diagram (schematic representation of international 10–20 system) indicating approximate locations of the cathode (blue) and anode (red) used for DC stimulation (B). Graphical representation of the effect of tDCS on the patient's seizures in a single session (C). The total number of myoclonic jerks was counted for each 15‐s epoch of video‐EEG and the mean seizure count for four consecutive epochs calculated (jerks/minute) and plotted (error bars are standard deviations). The solid bar indicates when tDCS (2 mA; 20 minutes) was applied. The data in (C) are further summarized in (D). In the bar graph, each bar plots the mean seizure frequency (jerks/second) before, during, and after tDCS (error bars represent 1 standard deviation from the mean). Bonferroni corrected t tests confirmed the significant effect of tDCS on seizure activity during tDCS. Posttreatment EEG (E), which confirmed that the seizure activity had ceased but some degree of encephalopathy continued. Note the change of EEG montage.

The location for the cathode was determined on the basis of the stroke‐like lesions identified on the serial MRI head (Figs. 1A,D) and the electrode locations on the pre‐tDCS EEG at which phase‐reversal of the epileptiform discharges occurred (see Fig. 2A). In the case described, the location approximated to T6, and therefore the cathode was placed over the right occipito‐tempero‐parietal region (centered on P4/T6; see Fig. 2B). The anode was applied to the left forehead (on a point approximating FP1). FP1 was chosen primarily as the location for the anode because it was contralateral and sufficiently anterior to the area of cortical edema (and the epileptogenic focus), thus reducing the possibility of causing an increase in seizure activity. The long axis of both electrodes was oriented in a coronal plane. tDCS was delivered by a battery‐powered (9V battery; IEC 6LR61) constant current stimulator (custom‐built by the Medical Physics Department, Newcastle upon Tyne Hospitals) through a pair of 5 × 7 cm conductive rubber electrodes covered in saline‐soaked sponges (neuroConn, Ilmenau, Germany).

To maximize the therapeutic effects of direct current stimulation, a stimulus intensity of 2 mA was administered for 20 min. To comply with the guidance of the NuTH NIPC, the stimulation parameters chosen (2 mA and 20 min) were deemed to be the maximum safe parameters used in children based on published literature.^6^

## Results

The frequency of seizures was reduced significantly during tDCS but returned to previous levels after tDCS (see Figs. 2C,D). However, after 3 days of treatment, the clinical seizures stopped altogether with the termination of ictal discharges on the EEG (Fig. 2E). A month later, the patient developed irregular twitching of abdominal muscles and lower limbs, which her EEG captured as a clear buildup of rhythmical sharp activity over the posterior quadrant of the right hemisphere. We applied a 14‐day course of tDCS using the same protocol. She reported no side effects other than a mild tingling or burning sensation under the electrodes during the stimulation. Her AED treatments were not adjusted during the period of tDCS treatment.

The patient was discharged home after a total of 4 months of hospitalization, and she received a prolonged period of intense physical rehabilitation in the community. At the most recent clinic review (12 months after discharge from the hospital), she had returned to her studies with additional educational support at school. She reports an unsteady gait but can walk unaided. She has intermittent, brief sensory seizures affecting the right side. Her AED regime includes levetiracetam 1,750 mg twice daily, phenobarbitone 90 mg twice daily, and perampanel 8 mg once daily. Clinical examination showed subtle myoclonic jerks, poor visual acuity bilaterally, restricted upgaze, astereognosis on the right, areflexia, and an ataxic gait.

## Discussion

Our patient's clinical presentation and neuroimaging changes are consistent with stroke‐like episodes driven by protracted seizures. The clinical management of acute symptomatic seizures proved extremely challenging, despite administration of multiple AEDs and general anesthetic agents, including thiopentone. However, we have demonstrated that the adjunctive use of tDCS successfully terminated the debilitating seizures. To our knowledge, this is the first successful application of tDCS in the refractory focal epilepsy caused by recessive *POLG* mutations.^5^

Transcranial direct current stimulation is a noninvasive subthreshold method of modulating cortical excitability using weak currents. First applied to the treatment of psychiatric disorders,^7^ there is now increasing interest in its potential therapeutic application to a range of neurological disorders, including focal epilepsy.^8^ The effects of tDCS on cortical excitability and the persistence of these effects are dependent on the current density applied (i.e., current intensity and electrode size), the polarity of the stimulus (cortical excitability is reduced by cathodal stimulation and increased by anodal stimulation), and the duration of DC stimulation.^9^ Although the clinical behavior of seizures observed in the context of mitochondrial diseases would suggest very different underlying mechanisms of epileptogenesis compared with other acquired or genetically determined epileptic syndromes, mitochondrial energy failure in neurones and glia is likely to be an important contributor. Cathodal tDCS will, as in all seizure disorders, reduce the probability of sodium and calcium channel opening and thus the probability of action potential generation (and seizure propagation) and, via long‐term depression (LTD), reduce the connectivity in epileptic networks.

Other putative mechanisms of action of tDCS include depression of NMDA‐receptor‐mediated synaptic signals, reduction of presynaptic inputs via postsynaptic hyperpolarization, and alterations in NMDA receptor efficacy and transmembrane protein migration.^10^, ^11^ However, it should also reduce the energetic demands on mitochondria by indirect effects on spiking activity and possibly by direct effects on glia^12^ and intracellular bioenergetics.^13^, ^14^, ^15^ To date, several studies involving patients with refractory focal epilepsy have provided some compelling evidence for the efficacy of tDCS in reducing the frequency of clinical seizures.^8^, ^16^, ^17^ The technique of tDCS is emerging as a promising noninvasive, nonpharmacological, adjunctive treatment for refractory epilepsy owing to its low risk profile,^8^ low cost, and ease of use compared to other surgical neurostimulation techniques such as deep brain stimulation and vagus nerve stimulation.

In summary, we report that tDCS was an effective adjunctive therapy in a teenager who presented with refractory *POLG*‐related focal motor status epilepticus. Given the high prevalence of epilepsy in patients with mitochondrial disease,^2^ we suggest that the application of tDCS in mitochondrial epilepsy warrants further evaluation in prospective clinical studies.

## Author contribution

Conception and design of the study: Y.S.N., R.M., and M.R.B. Acquisition of data: All authors. Analysis and interpretation of data: Y.S.N., H.V.R., M.L., V.R., R.W.T., D.M.T., G.S.G., R.M., and M.R.B. Drafting the manuscript or figures: Y.S.N., H.V.R., R.M., and M.R.B. Critical review and revision: All authors.

## Disclosure

Nothing to report. We confirm that we have read the Journal's position on issues involved in ethical publication and affirm that this report is consistent with those guidelines.

## Supporting information


**Figure S1.** Neuroimaging and electrophysiology.

**Data S1.** Supplementary data.
