# Supplementary figures and images for: The adjunctive application of transcranial direct current stimulation in the management of de novo refractory epilepsia partialis continua in adolescent‐onset POLG‐related mitochondrial disease
Source: Epilepsia Open. 2018 Jan 11;3(1):103–8. doi: 10.1002/epi4.12094 (PMC5839316; doi:10.1002/epi4.12094)

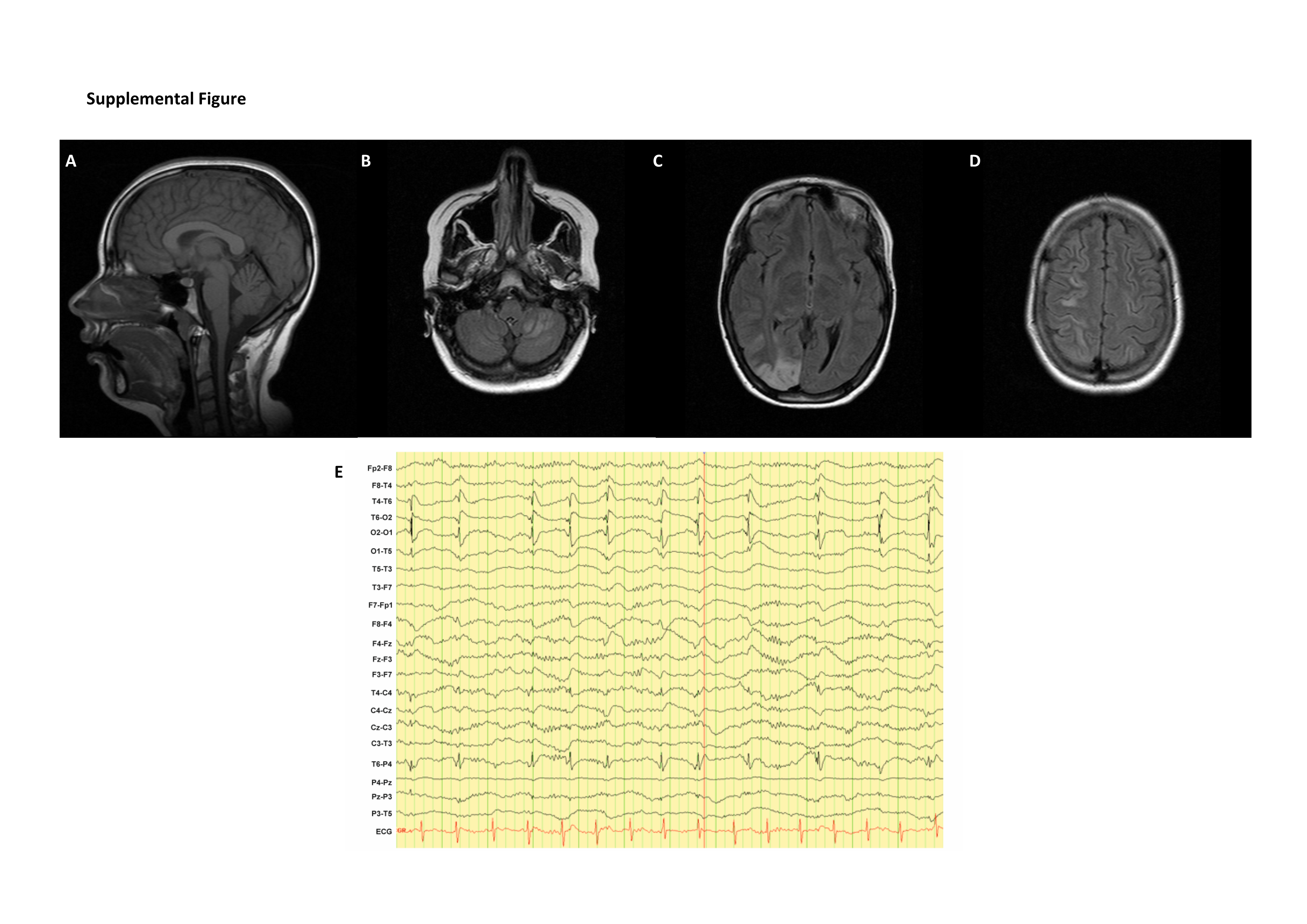

Supplement: Supplementary file 1 — Figure S1. Neuroimaging and electrophysiology. [file EPI4-3-103-s001.tif]
