# Supplementary material for: The adjunctive application of transcranial direct current stimulation in the management of de novo refractory epilepsia partialis continua in adolescent‐onset POLG‐related mitochondrial disease
Source: Epilepsia Open. 2018 Jan 11;3(1):103–8. doi: 10.1002/epi4.12094 (PMC5839316; doi:10.1002/epi4.12094)
Supplement: Supplementary file 2 — Data S1. Supplementary data. [file EPI4-3-103-s002.docx]

**Supplementary data**

**Case 1**

A 17-year old, right handed college student was referred to the neurology clinic due to a progressive clumsiness that began around age ten. She also reported slurred speech and mild cognitive difficulties. There was no family history of ataxia or other neurological diagnosis. She was a non-smoker and consumed alcohol occasionally. Clinical examination revealed mild myoclonus, cerebellar signs including fine horizontal nystagmus, dysarthria, incoordination of upper limbs, ataxic gait and generally depressed reflexes. Laboratory investigations including full blood count, kidney and liver function tests, coeliac screen, auto-antibodies, serum vitamin B12 and folate were within normal limit. Mild cerebellar atrophy was evident on the MRI head (**Supplemental Figure 1A**). Cerebrospinal fluid (CSF) analysis was unremarkable except mildly raised protein 0·61g/L (normal <0·45g/L).

Three months later, she was admitted with right focal motor seizure that evolved to bilateral convulsion and respiratory arrest. The cause of seizure was initially attributed to the recreational use of amphetamine and the seizures were self-limited on the first admission. She represented with increased confusion, intermittent positive visual phenomena and right arm twitching less than a week later. Her electroencephalogram (EEG) showed changes compatible with non-convulsive status epilepticus, with an active focus on the left occipital-temporal region, which correlated with the imaging changes. Her seizure control was achieved with a combination phenytoin, levetiracetam and clobazam. She subsequently underwent a muscle biopsy that showed two percent of cytochrome *c* oxidase-deficient fibers and minor mitochondrial DNA rearrangement. Sequencing of *POLG* gene identified compound heterozygous mutations, p.Ala467Thr and p.Trp748Ser, in the linker region.

Her general condition deteriorated following the first episode of status epilepticus. She developed dysphagia and break-through focal seizures frequently precipitated by urinary tract infections. She became wheel-chair dependent due to a combination of sensory ataxia (sensory ganglionopathy shown in nerve conduction studies) and cerebellar ataxia. At age 19, she was admitted with a further prolonged episode of status epilepticus, of which she was treated with multiple anti-epileptic drugs (AEDs) at maximal doses and attempted burst suppression with thiopentone. Repeat head MRI showed new changes in the left cerebellar hemisphere, right occipital-parietal cortices and right motor strip (**Supplemental Figure 1B-D**). Serial EEGs showed migratory ictal foci and encephalopathy (**Supplemental Figure 1E**). Generalized seizure was controlled with medications but the epilepsia partialis continua (EPC) persisted for three weeks. She unfortunately died from severe sepsis and multi-organ failure.

**Supplemental Figure Legend**

**Supplemental Figure 1. Neuroimaging and electrophysiology.** Sagittal T1-weighted MRI of head showed mild cerebellar atrophy at age 17 (A). Subsequent MRI head was performed when patient presented with a status epilepticus at age 19. FLAIR sequence showed hyperintensities in the left cerebellar hemisphere (B), right occipital cortex (C), right frontal-parietal region (D) with restricted diffusion. EEG showing continuous seizure activity over the right parieto-occipital region (E).
